# A Modern Diagnostic Procedure—The Introduction of Point-of-Care Ultrasound in Romanian Emergency Physicians’ Daily Routine

**DOI:** 10.3390/clinpract14030090

**Published:** 2024-06-14

**Authors:** George-Catalin Bouros, Tudor Ovidiu Popa, Paul Lucian Nedelea, Emilian Manolescu, Anca Haisan, Iulia Roca, Petruta Morosanu, Alexandra Hauta, Gabriela Grigorasi, Mihaela Corlade-Andrei, Diana Cimpoesu

**Affiliations:** 1 Department of Emergency Medicine, “Grigore T. Popa” University of Medicine and Pharmacy, 700115 Iasi, Romania; bouroscatalin@yahoo.com (G.-C.B.); paul.nedelea@yahoo.com (P.L.N.); manolescu.emilian@gmail.com (E.M.); anca.haisan@umfiasi.ro (A.H.); iulia.roca@umfiasi.ro (I.R.); morosan.petruta@gmail.com (P.M.); alexandra.hauta@umfiasi.ro (A.H.); gabriela.tiulica@yahoo.com (G.G.); carmen.cimpoesu@umfiasi.ro (D.C.); 2 Emergency Department, “St. Spiridon” Emergency Clinical County Hospital, 700111 Iasi, Romania

**Keywords:** ultrasound, point-of-care, emergency department, education

## Abstract

Background: Emergency medicine in Romania has developed fast since inception. The need for faster diagnostic capabilities due to the high workload pre- and in-hospital made point-of-care ultrasound (POCUS) a logical next step. The advantages of POCUS are well known, but implementation presents challenges. Our goal was to study how a straightforward method of implementation would work locally. Methods: Two prospective observational studies were conducted at 6 months (prehospital) and 4 months (in-hospital). The protocol used was extended focused assessment sonography in trauma (eFAST), and the shock index (SI) was used to stratify patients. Voluntary sampling was conducted by emergency physicians. The primary outcomes were patient numbers, type of case use, results, and accuracy. Results: The prehospital study registered 34 patients: 41% traumas, 35% cardiac arrest, 18% shock, and 6% acute respiratory distress. The in-hospital study patients were 78: 36% traumas, 6% cardiac arrests, 41% shock, and 17% acute respiratory distress. A total of 88.5% of the cases were confirmed with definitive imagistic findings. Conclusion: The studies mark an increase in POCUS usage and use in complicated cases. Providing supervision and feedback into clinical practice resulted in a further increase in POCUS usage, the second study having an 88.5% accuracy when compared to the final diagnostic proving the increased efficiency of a longitudinal training approach.

## 1. Introduction

The development of ultrasound has had a long history from its inception to its ascent from bulky one-room machines to the point-of-care ultrasound (POCUS) status it has now. Images used to be hard to obtain and harder to interpret but with the advancement of technology, obtaining images has become easier, cheaper, and thus more accessible [1]. Due to time constraints and the inaccessibility of a fast, repeatable diagnostic imaging technique, a way to answer acute questions was needed, and POCUS matched these requirements. It was initially adopted within fast-paced clinical settings in the United States in the 1990s and later introduced in the resident training curriculum [2,3].

Romania does not provide an ultrasound curriculum for emergency medicine trainees; major emergency departments have radiologists working with emergency physicians to provide efficient patient care [4]. The radiology team interprets ultrasound, X-ray, and emergency computer tomography (CT) results, but due to an increase in workload, the severity of cases and the high addressability to the emergency department, a way to optimise triage for advanced imaging needs to be implemented [5]. Introducing POCUS as a standard tool for the emergency physician would lead to improved patient care, triage the need for further imaging, and reduce waiting times [6].

Iasi, Romania, has an official population of 500 thousand people and close to 1 million in the metropolitan area. Our emergency department at St. Spiridon Hospital is a level 1 trauma centre—it has close to 100,000 presentations per year. This hospital is the main training centre for emergency medicine, anaesthesia, intensive care, and many other specialities in the region, making it an ideal centre to implement new curricula and change ways of caring for patients.

Our aim is to build up familiarity with ultrasound use and confidence by providing point-of-care ultrasound courses and workshops, designed around eFAST, abdominal aortic aneurysm, lung, and vascular access ultrasound protocols. We have been holding these courses yearly since the first half of 2019 in collaboration with the European Society for Emergency Medicine and evaluating its use in clinical practice in the pre-/in-hospital environment for the emergency physicians working in our department. This being the first study of ultrasound implementation in our region for emergency medicine, we sought to provide ultrasound as a clinical support tool in pre-/in-hospital and to verify in hospital accuracy of POCUS findings in comparison to diagnostic imaging techniques, understanding particularities of use and development needs for established and training physicians, in order to implement ultrasound in daily activities for current and future doctors [7].

## 2. Materials and Methods

### 2.1. Study Aim

The aim was to observe the effects yearly ultrasound workshops would have on the clinical practice of emergency physicians in the emergency department and prehospital and if this would be an initial viable alternative to a curriculum. We followed frequency of use, patient selection, case severity, and result accuracy. POCUS use was encouraged as a clinical support adjunct and not as a definitive diagnostic.

Our second objective was to understand the opinion of local established medical personnel on POCUS use and its implementation in daily practice where, currently, most emergency physicians seldom use it and do not follow a structured approach.

### 2.2. Study Setting

Two prospective observational studies were conducted with collaboration from the prehospital and ED teams of the St. Spiridon University Hospital in Iasi, Romania, between January 2022 and September 2023. The department has a team of 23 doctors who practice prehospital and in-hospital duties, working in shifts of 12 or 24 h.

Our hospital manages two prehospital crews, one land and one helicopter crew (HEMS); the latter was not included in current studies. The land ambulance crew managed by our institution has a permanent physician as part of the team with an average number of cases between 3 and 5 per 24 h; some missions are interhospital transfer of critical patients. The ED has between 200 and 300 patients per 24 h, and peaks may vary; 15 beds are available for acute presentations. Patients are examined according to initial triage in either minor, major, or resuscitation zones.

Ultrasound scans conducted by emergency physicians in this study were done for patients that were coded for the resuscitation zone and seldom for major or lower triage cases, using a GE Healthcare Vivid T8 (General Electric Company, New York, NY, USA). The prehospital ambulance crew has a portable, pocket-sized ultrasound outfitted with the GE Handheld VScan Dual Probe (General Electric Company, New York, NY, USA).

Ethics commission approval was obtained from the University of Medicine and Pharmacy Grigore T. Popa Iasi and the ethics commission board of the Iasi St. Spiridon Hospital, number 147/31.01.2022.

### 2.3. Workshop Design

POCUS courses have been held since 2019 with the collaboration of European certified bodies. These are structured over a two-day period with participants arranged in groups that participated in themed workshops, prioritising hands-on experience with experienced instructor supervision, and dedicated scanning time for each participant.

Workshop design included different stations, each with a different theme, covering ultrasound basics, eFAST, abdominal, aorta, lung, and cardiac views complementary with intravenous access workshops and case simulations to integrate all new skills at the end of the two days. Ultrasound volunteers with different pathologies, which would benefit the learning experience, were asked to participate. Lectures were held before each major hands-on workshop, dedicated to covering important aspects to be taught. Gatherings and live questionnaires using online interactive presentations software such as mentimer.com were used to understand participant shortfalls, discuss, and guide the approach according to the level of knowledge and usage trends, and identify challenges for training physicians.

### 2.4. Study Design

Emergency Medicine Specialty Training in Romania prepares doctors for prehospital medicine in parallel with department training starting with the second year of residency (5-year training program). With the goal of making ultrasound available in all operating environments, we introduced it to the prehospital crews in 2020. With the debut of our study, we investigated usage trends, identified issues the doctors were facing, addressed them, and then conducted a similar in-hospital study. For the in-hospital study, we cross-checked results with the radiology team (diagnostic ultrasound and computer tomography results).

The main cases our land crew are presented with are critical care cases, so we categorised result reports into patients that fit one of the following groups: 1. shock, 2. trauma, 3. cardiac arrest, and 4. acute respiratory distress. The same triaging and patient selection were used for the in-hospital study as well. The scanning protocol used in both studies was eFAST, which is useful in detecting abdominal, lung, and cardiac findings.

We used the shock index (SI) to estimate the severity of cases, and a cutoff value of 0.75 SI was considered severe. “This is defined as the heart rate (HR) divided by systolic blood pressure (SBP). It has been studied in patients either at risk of or experiencing shock from a variety of causes: trauma, haemorrhage, myocardial infarction, pulmonary embolism, sepsis, and ruptured ectopic pregnancy. An SI > 1.0 has been widely found to predict increased risk of mortality and other markers of morbidity such as massive transfusion protocol activation and admission to intensive care units” [8].

The ultrasonic examinations performed by the physicians, either pre- or in-hospital, were documented in a standard report form, documenting demographic causes for examination, hemodynamic parameters, sonographic expectations, findings, diagnosis, management, elapsed time, and difficulties encountered. The physicians completed the reports voluntarily and submitted them anonymously. For the in-hospital study, the reporting physicians were also asked to report the final diagnosis and definitive imaging results. Evidence-based clinical analysis was used to understand final outcomes.

Cross-sectional studies, in the form of questionnaires before and between the studies, identified the status of interest and hindrances in the use of ultrasound. The questionnaires were made available electronically and distributed via email, with results registered anonymously.

### 2.5. Selection of Participants

**Figure 1:**
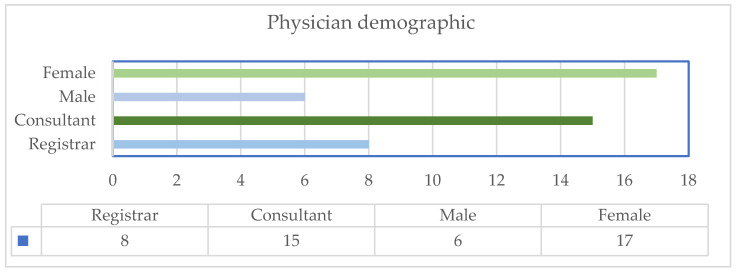
Emergency physicians involved in study demographics.

Voluntary response sampling was done by the registrars and consultant-level physicians, who perform prehospital and in-hospital duties in our department (Figure 1). Physicians who participated had attended at least one previous POCUS course organised or recognised by our institution. The number of physicians participating in the second study decreased after some voluntary withdrawal.

### 2.6. Data Collection and Measurements

The performing physician reported the following information gathered from ultrasounds: demographics, cause for examination, time from call to the patient, time from contact to ultrasound, time for ultrasound protocol duration, haemodynamic parameters, the targeted anatomical region, and results obtained. In the in-hospital study, the definitive result was also asked to be submitted to verify accuracy.

### 2.7. Statistical Analysis

The data was collected and analysed in IBM SPSS version 20 (IBM, New York, NY, USA). The qualitative data was described using frequencies (absolute values) and percentages. A point-biserial correlation was employed between qualitative data and quantitative data, whereas the difference between two qualitative data was assessed by the Chi-square test. All statistical tests were significant at a threshold of *p* < 0.05.

## 3. Results

The baseline for the study was 2019. A simple questionnaire was anonymously carried out between the participants of the first ultrasound course. A total of 21 participants answered our call out of 40 that attended; this resulted in a response rate of 52.5%. For most of the participants, this was the first ultrasonography course they attended, but 28.7% had attended US courses previously.

The participants were 66.66% registrars, 28.58% consultants, and 4.76% junior doctors. Even though they were without previous formal training, 71.42% were already using US in their practice. Ultrasound was used to check for cardiac activity in pulseless electrical activity (PEA) (76.19%), detect free intraabdominal fluid in trauma (57.14%), and facilitate invasive procedures such as central venous line placement and pleural taps (Table 1. A net increase in usage rates and trends of US use were reported during the follow-up questionnaire, but second opinions were very common, and no major decisions were made with only initial operator scanner findings.

Prehospital Study: This study took place between January 2022 and June 2022 with 23 emergency physicians. A total of 34 patients were reported. The shock index was used to determine severity. Patients’ info was anonymised and divided into four categories after initial triage: 1. 18% shock, 2. 41% trauma, 3. 6% cardiac arrest, and 4. 35% acute respiratory distress. The shock index was used to determine severity. The median age was 57, with the minimum at 18 and the maximum at 81. The female-to-male ratio was 2.1:1. After primary evaluation, patients were divided, as shown in Table 2.

(1) Shock—Six patients: two patients had an undifferentiated shock, 0 were septic, four had a cardiac shock, and none had an obstructive shock. A shock index of >0.75 was found in four patients. Ultrasound findings reported were four patients with hypokinesia and two with right ventricle (RV) strain.
(2) Trauma—14 patients: 12 due to road traffic accidents and two falls from a height (more than two meters). Of the 14 patients with trauma, eight patients had an SI > 0.75. eFAST scans were carried out and six patients were positive for abdominal free fluid and two for pneumothorax.
(3) Cardiac arrest—12 patients had the following initial rhythm: eight asystole, two pulseless electrical activity, and two with ventricular fibrillation or ventricular tachycardia. On ultrasound scan during CPR, our teams found three patients had mechanical activity, and two presented with right ventricle strain.
(4) Acute respiratory distress—two patients, both with an SI > 0.75 but with no significant findings on the ultrasound scan.

Intermediate anonymous survey: The first ultrasound study presented a lot fewer results than expected. We decided to hold a meeting with the doctors involved and discuss issues that came up. To facilitate an open and transparent discussion, an anonymous 11-question survey was run prior to the meeting.

All 23 participating physicians answered within the allotted time. Fourteen specialist doctors and nine consultants—level 5 (21.7%) (three consultants and two registrars) stated that they prefer not to use POCUS prehospital because of the lack of experience required to perform POCUS safely under acute settings and opted out of a further study. A worry about a lack of experience with POCUS was also stated by the doctors who opted to keep participating in the study. Eighteen (100%) said they use it for trauma and cardiac arrest patients, 14 (77.8%) use it in nondifferentiated shock, and 11 (57%) use it for invasive procedures. Sixteen (88.89%) have used POCUS for the evaluation of pulseless electrical activity. All the participants consider that POCUS has increased in clinical importance since the start of the pandemic, and 10 (55.56%) state that the use of POCUS changed the management of their patients, reporting an improvement in care decisions. Eighteen (100%) concluded that bi-annual workshops would be better suited for skill development and maintenance and that more support for learning POCUS users is needed. All (100%) of them also said they felt strongly about introducing ultrasound in the training curricula for future residents.

Following the experience of the first study, we reshaped the workshops offered by our department, introducing local lecturers from the radiology and cardiology departments of our hospital. Conducting workshops with ultrasound specialists who work within the same, or close to, the environment as the emergency physicians led to better communication, real-time feedback, and a safer environment to learn during actual live patient usage.

**Figure 2:**
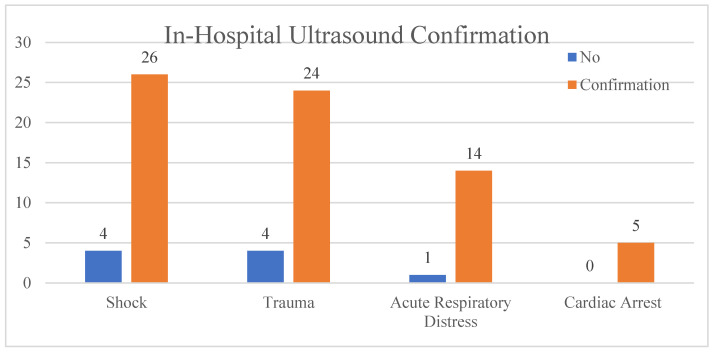
In-hospital ultrasound confirmation.

In-Hospital Study: This study took place between June 2023 and September 2023, with 18 physicians. A total of 78 scans were registered. The shock index was used to determine the severity of the examined patients. Patients that resulted from this study were anonymised, and results were divided into four categories after primary evaluation: 1. 41% shock, 2. 36% trauma, 3. 6% cardiac arrest, and 4. 17% acute respiratory distress (Table 3). A total of 88.5% of ultrasounds were confirmed by definitory investigations (Figure 2).

(1) Shock—32 patients: 18 cases of undifferentiated shocks, four septic, eight cardiac shock, and two cases with obstructive shock. Seventeen out of the 32 patients had an initial shock index of over 0.75. The median age was 59.57, with a minimum of 20 and maximum of 90, with a female: male ratio of 1:1.2. Abdominal scans showed three positive findings for fluid and one for ectopic pregnancy, one positive chest scan for fluid, nine cardiac scans with hypokinesia, and four positives with RV strain. Findings were confirmed in 84% of scans carried out, and 16% were informed by further formal imaging.
(2) Trauma—28 patients: nine road traffic accident passengers, two pedestrians, 12 falls from height (six patients from more than 2 m), and five physical aggressions (one stab patient and four blunt trauma). Eleven out of 28 patients had a shock index of >0.75. The median age was 53, with a minimum of 21 and a maximum of 90 and a female-to-male ratio of 0.8:2. All 28 patients had eFAST scans: two were positive for abdominal fluid, one haemothorax, and two pneumothorax—confirmation of 86% and 14% informed by further formal imaging.
(3) Cardiac arrests—five patients: four patients with asystole, one with pulseless electrical activity, and 0 with ventricular fibrillation/pulseless ventricular tachycardia. One scan with a positive finding for RV strain and one chest scan was positive for fluid. Confirmation was done by filming scans and rechecking with 100% confirmation.
(4) Acute respiratory distress—13 patients: nine undifferentiated causes, two septic patients, and two chronic patients with flare-ups. A shock index of >0.75 was identified in six out of 13 patients, the median age was 71, with a minimum of 50 and a maximum of 91. One positive abdominal scan for fluid, seven positive chest scans for pleural fluid, one with hypokinesia, two RV strains, and three scans positive for pericardial fluid. All scans were confirmed with 100% accuracy.

According to the Pearson correlation coefficient, no statistically significant correlation has been identified between the shock index and the ultrasound confirmation in the in-hospital phase (r = −0.04, *p* = 0.73).

**Figure 3:**
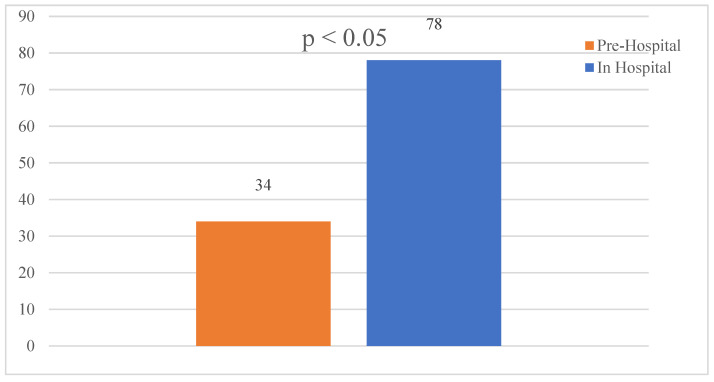
Prehospital vs. in-hospital reported cases over 181 and 121 days, respectively.

**Figure 4:**
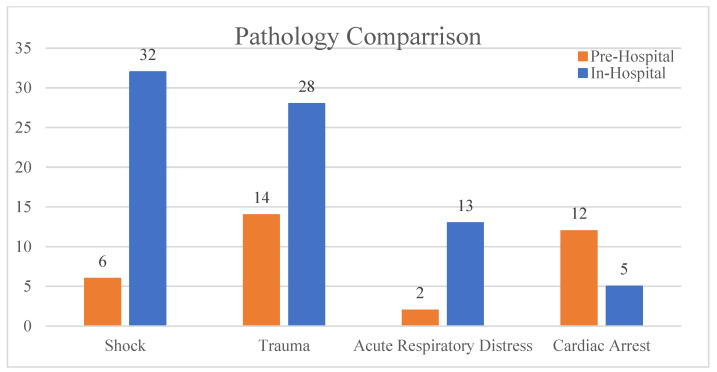
Prehospital vs. in-hospital reported cases based on initial triage.

A chi-square test for independence was conducted to examine the differences between Study 1 and Study 2 in terms of the number of cases reported, the number of doctors, and the days elapsed, yielding a significant result (*p* < 0.05) (Figure 3). A comparison of triaged cases from both studies shows a marked increase in the initial presentation of shock and trauma but a decrease in cardiac arrest cases that had POCUS performed compared to the prehospital study (Figure 4).

According to the Pearson correlation coefficient, no statistically significant correlations have been identified between the shock index and any ultrasound findings: abdominal (r = 0.07, *p* = 0.45), pulmonary (r = −0.18, *p* = 0.08), and cardiac (r = −0.04, *p* = 0.68).

## 4. Discussion

The aim is to implement POCUS in the daily practice of current and training generations of emergency physicians that are being trained at our centre. The literature on this subject in this geographical area is scarce, and this case study provides a glimpse of the effort to implement ultrasound techniques in any other specialty than the traditionally ultrasound-heavy ones, such as radiology, cardiology, obstetrics, and gynaecology. The term point-of-care ultrasound originated in Emergency Medicine, and it refers to ultrasound where it is needed, tightly knit with patient evaluation, management, and treatment [9]. Its goal is to answer simple questions by confirming or infirming medical suppositions: the presence of abdominal fluid? Presence of ectopic pregnancy? Is there an abdominal aortic aneurysm? Yes or no questions specific to the patient being evaluated [10].

Ultrasound has become globally accessible. It had a big increase in quality, with artificial intelligence (AI) technology, and a decrease in cost and size in recent years, making it easier to implement in resource-scarce environments and developing parts of the world [11,12]. We find ourselves at a crucial moment in POCUS development due to ease of access—this comes with a risk due to a lack of a generally accepted standard of training and, thus, difficulties in maintaining quality standards with patient care [13]. POCUS is highly dependent on user skill, and insufficient knowledge can lead to misdiagnosis [14,15].

POCUS usage started a lot earlier in the United States and it was clear since the early 1990s that a structured curriculum is needed. In 2001, the American College of Emergency Physicians (ACEP) approved the first comprehensive guidelines for ultrasound use for emergency medicine [16]. Since then, numerous organisations have adapted an ultrasound training curriculum based on the ones released by ACEP. In the last few years, more organisations from around the world have started to bring forward the need not just for POCUS education but a need to ensure governance, infrastructure, administration, and quality assurance processes are put in place to support education for POCUS [17].

In 2023, the European Union Society for Emergency Medicine (EuSEM) and the European Federation of Societies for Ultrasound in Medicine and Biology (EFSUMB) proposed the implementation of European Guidelines that would set a standard for European POCUS stewardship to cater to the different needs encountered in different settings around Europe and combat current faulty practices [13].

Locally, POCUS has become more focused during the COVID-19 pandemic. This started to put pressure on the traditional tight collaboration of acute medicine and diagnostic radiological investigations due to contamination risks. Even simpler hands-on manoeuvres, such as using the stethoscope to listen for pathological lung sounds or check for proper intubation, became difficult due to safety requirements and the use of personal protective equipment (PPE). Documented efforts to standardise POCUS usage and encourage proper use under pandemic conditions are seen in national training centres, proposing the introduction of ultrasound to counter imagistic and accessibility deficiencies [18].

The novelty of our study is the documentation of POCUS use as an adjunct in enhancing emergency physician evaluation capabilities in the Romanian prehospital environment and in the emergency department to help guide resuscitation or the need for further imagistic investigations. We came to the same conclusion as many before us that a straightforward approach is insufficient to implement POCUS as a standardized technique successfully and that uniformisation of training results requires attention to the different learning styles of students [19,20]. This was hinted by the modest results of the prehospital study where the doctors involved concluded that, despite the multiple training sessions carried out, they felt unsure about performing POCUS and making decisions based on their findings in certain time-sensitive situations and preferred not to use it.

The statistically significant increase in cases in the second study is due to multiple changes: a change in environment, a change in the faculty that participated in the training sessions, and support provided thereafter. The initial thought was that off-site faculty would provide better learning conditions for our participants due to a more objective approach from both sides, but we did not account for the lack of continuity this would imply. We started collaborating with an on-site faculty from the cardiology and radiology departments, and this brought a positive change in interdepartmental communication. It created a more appropriate environment to learn and develop POCUS knowledge while facilitating collaboration, and experience exchange between doctors and a continuation of support for POCUS development.

The 88.5% confirmation rate of ultrasound in the in-hospital study showed that the ultrasound training sessions provided the trainees with the ability to recognise and interpret images for acute situations accurately—this result is comparable with other studies documenting the same type of study of 90.8% and 94.46% [21,22]. The first study outlines that ultrasound training should not be limited to training workshops and should be extended through to clinical practice in order to acquire experience and confidence in skill usage in acute situations. This requires trainees to have access to skilled and willing supervisors or risk having them not follow through after the initial introductory ultrasound course [13]. The courses have eased the way to the implementation of extracorporeal cardiopulmonary resuscitation (ECPR) in our department, as per the latest resuscitation guidelines—this translates to highly qualified and confident users of POCUS, but the aim must be the uniformisation of ultrasound use among physicians and not just for a highly qualified team [23].

Although the experience thus far concludes the need for further changes to safely standardise this technique locally, a major step forward has been made in the form of establishing future confident and eager POCUS mentors in our emergency department. This initial hurdle of creating mentors is almost traditional, as seen in previous experiences of research in emergency medicine where the need to find mentorship and development of advanced skills was done through fellowships with other experienced researchers in other specialities [24].

Having made the initial steps of forming local mentors, further studies that assess the true impact of POCUS use by emergency physicians on patient care, and not just the accuracy of aetiology identification, need to be done. These should be done in conjunction with new educational directions derived from the lessons learned through this article and in collaboration with national and European institutions. [13,25]. Level 1 trauma centres in Romania manage permanent HEMS crews, and successful implementation of a POCUS curriculum could impact patient care and decisions of transfer for patients to specialised centres from rural, mountainous, isolated, or low-resource areas.

## 5. Limitations

1. Underreporting of cases: This was more evident in the prehospital POCUS study as the number of cases reported was less than expected.
2. The prehospital study presented difficulties in comparing operator results to a standard ultrasound or CT investigation as many patients succumbed to their injuries before successfully being transported to the hospital or were not followed up on by a submitting physician.
3. Incomplete timelines on acquisition report sheets: Not documenting the duration of ultrasound scans could indicate that either reports were being completed much later after the case was resolved and the exact timelines were not recalled correctly but could also hint that the ultrasound protocol conducted was incomplete, was conducted improperly, or was taking longer than expected due to unmentioned difficulties encountered.
4. Maintenance of previous ultrasound use tendencies despite participation in courses and workshops: This was expected, since many of our users are self-taught and have been using ultrasound in their practice, having learned from various sources. The addition of supported workshops and courses has been beneficial but not enough to replace ingrained behaviour.
5. Limited generalizability: The study has only been conducted in one university training centre for residents in the country out of nine available nationwide.
6. The overall low numbers submitted, given that the department sees 200–300 cases per day, is due to the fact that patients were seen in the resus room and not every shift was covered by physicians comfortable with POCUS.

## 6. Conclusions

Training sessions once or twice per year were not sufficient to create confidence for prehospital/time-sensitive use of POCUS in established emergency physicians—they preferred, in most situations, to rely on amnestic and clinical examination when resource constraints were present.

Providing trainees with supervision and feedback after initial training sessions extended into clinical practice resulted in higher POCUS usage and confidence, as shown by an increase in submitted results. This paired with the high accuracy of 88.5% achieved by comparing operator results with results from advanced imaging, proved the efficiency of a longitudinal training approach for POCUS users.

To further develop and uniformise POCUS use in acute settings for current and future trainees requires steps to be taken towards creating governance, infrastructure, administration, and quality assurance processes to support education. Without these, there is a risk of false reassurance or even misdiagnosis from incorrectly trained users. These steps can be achieved through collaboration with other national centres to coordinate resident training programs and implementation of the curriculum in partnership with European-certified bodies such as the European Union Society of Emergency Medicine (EuSEM) and the European Federation of Societies for Ultrasound in Medicine and Biology (EFSUMB) who are making strides to conglomerate these efforts.

## Figures and Tables

**Table 1:** Initial evaluation of ultrasound familiarity.

Characteristics	Numbers
Participants answered Registrars Consultants Junior doctors Previous US courses Used in cardiac arrests Used in trauma	21/40 (52.5%) 14 (66.6%) 6 (28.58%) 1 (4.76%) 28.7% 76.19% 57.14%

**Table 2:** Prehospital study results.

Category	Patients	Shock Index > 0.75	Causes/Initial Examination	US Findings
Overall	34	14		19 Positive findings
Shock	6	4	2 Undifferentiated, 2 Septic, 4 Cardiac,	4 Hypokinesia, 2 RV Strain
Trauma	14	8	12 Road Traffic Accidents, 2 Falls from Height	6 Free abdominal fluid, 2 Pneumothorax
Cardiac Arrest	12	N/A	8 Asystole, 2 PEA, 2 VT/VF	3 Mechanical activity, 2 RV strain
Acute Respiratory Distress	2	2	N/A	N/A

N/A: not applicable.

**Table 3:** In-hospital study results..

Category	Patients	Shock Index > 0.75	Causes/Initial Examination	US Findings	Accuracy
Overall	78	34		29 Positive findings	
Shock	32	17	18 Undifferentiated, 4 Septic, 8 Cardiac, 8 Obstructive	4 Hypokinesia, 2 RV Strain	27 Confirmed 5 Infirmed
Trauma	28	11	9 Road traffic accidents, 2 Pedestrians 12 Falls from height 4 Blunt trauma (aggression) 1 Stabbing (aggression)	2 Free abdominal fluid, 2 Pneumothorax 1 Haemothorax	24 Confirmed 4 Infirmed
Cardiac Arrest	5	N/A	4 Asystole, 1 PEA, 0 VT/VF	1 Pleural fluid, 1 RV strain	5 Confirmed 0 Infirmed
Acute Respiratory Distress	13	6	9 Undifferentiated RD, 2 Sepsis, 2 Chronic flare-ups	1 Free abdominal fluid, 7 Pleural fluid, 1 Hypokinesia, 2 RV strain 3 Pericardial fluid	13 Confirmed 0 Infirmed

N/A: not applicable.

## Data Availability

The data that supports the findings is available on request from the corresponding author.
